# Co-occurrence of GATA2 deficiency and probable constitutional mosaic STAG2 alteration in a young adult with myelodysplastic syndrome: a case report

**DOI:** 10.3389/fmed.2026.1935947

**Published:** 2026-09-02

**Authors:** Onda-Tabita Calugaru, Daniel Coriu, Cerasela Jardan, Claudia Tarniceriu, Daniela Cristina Anghel, Iulia Ursuleac

**Affiliations:** 1 “Carol Davila” University of Medicine and Pharmacy, Bucharest, Romania; 2 Fundeni Clinical Institute, Bucharest, Romania; 3 “Grigore T. Popa” University of Medicine and Pharmacy, Iasi, Romania; 4 Saint Spiridon County Emergency Hospital, Iasi, Romania

**Keywords:** GATA2 deficiency syndrome, germline, germline variant classification, myelodisdplastic/myeloproliferative disorders, somatic variant analysis, young adult

## Abstract

Germline predisposition syndromes are increasingly recognized in young patients presenting with myelodysplastic syndromes (MDS) and bone marrow failure. Among these, GATA2 deficiency represents a well-established cause of hereditary susceptibility to myeloid malignancies, whereas constitutional STAG2 alterations are rare and mainly associated with cohesinopathies and neurodevelopmental disorders. We report the case of a 25-year-old male presenting with hypocellular MDS, recurrent severe infections, lymphedema, cytopenias, psoriasis, and multiple dysmorphic features. The patient had previously received a clinical diagnosis of Emberger syndrome without molecular confirmation. Targeted next-generation sequencing identified pathogenic truncating variants in GATA2, STAG2, and ASXL1. The variant allele frequencies observed for GATA2 and STAG2, together with the patient's young age and syndromic phenotype, prompted additional testing using non-hematopoietic tissue. Analysis of buccal-derived DNA confirmed the germline GATA2 pathogenic variant and detected the STAG2 alteration at a variant allele frequency consistent with probable constitutional mosaicism. Cytogenetic studies excluded sex chromosome aneuploidy. The co-occurrence of GATA2 deficiency and probable constitutional mosaic STAG2 involvement raises the hypothesis that alterations affecting distinct hematopoietic regulatory mechanisms may contribute to the complex clinical phenotype; however, functional interaction between these alterations remains unproven. This case highlights the importance of comprehensive molecular evaluation and germline confirmation in young patients with MDS, particularly when clinical features suggest an underlying inherited predisposition or an atypical overlapping phenotype. It further illustrates the diagnostic challenge of distinguishing acquired from constitutional alterations and the importance of tissue-specific molecular assessment when constitutional mosaicism is suspected.

## Introduction

Myelodysplastic syndromes (MDS) and hematologic neoplasms are still considered rare diseases, and according to European Leukemia Net Guideline (ELN 2022), Acute Myeloid Leukemia (AML) and MDS may occur in childhood and young adulthood in the context of multiple cancer predisposition syndromes. Among these, GATA2 deficiency (OMIM #137295) can be distinguished by its highly specific clinical phenotype.

GATA2 deficiency represents a clinically heterogeneous germline predisposition syndrome with variable penetrance and a high lifetime risk of myeloid malignancies, often posing diagnostic challenges due to incomplete or atypical phenotypic expression. In contrast, STAG2 alterations display a dual biological role: while germline variants are rare and primarily associated with neurodevelopmental disorders such as Mullegama-Klein-Martinez syndrome (MKMS), somatic mutations are frequently observed in myeloid malignancies and are considered markers of clonal evolution and disease progression (1–3).

Germline mutations in the GATA2 gene lead to one of the best-characterized groups of hereditary predisposition disorders, manifesting as Emberger syndrome, MonoMAC syndrome, or immunodeficiency with variable expressivity, and confer a high lifetime risk of MDS. Clinical suspicion often arises in childhood due to lymphedema, recurrent infections, or cytopenias, while confirmation relies on genetic testing. Importantly, a significant proportion of patients may remain undiagnosed or underdiagnosed, particularly when initial manifestations are limited to isolated monocytopenia or mild bicytopenia. In such cases, the underlying constitutional predisposition may remain unrecognized at the time of MDS diagnosis, potentially leading to incomplete etiologic characterization and management without consideration of the inherited disorder (4–9).

In pediatric patients in particular, cytopenias require a multidisciplinary evaluation, including careful clinical genetic assessment, as dysmorphic features may be subtle and easily overlooked. Therefore, extended genetic testing should be considered even in cases where the clinical phenotype is not strongly suggestive of a syndromic condition. During disease evolution, patients with GATA2 deficiency are known to develop and acquire somatic mutations characteristic of MDS progression, such as STAG2, ASXL1, SF3B1, TET2, U2AF1, among others (10–12).

In contrast, germline cohesinopathies caused by mutations in STAG2, RAD21, SMC1A, and SMC3 are rarely reported outside the field of neurodevelopmental disorders. Germline STAG2 variants underlie MKMS (OMIM #301022), an X-linked disorder characterized by dysmorphic features, developmental delay, and, in some cases, hematologic abnormalities (13–15). Patients with mosaic STAG2 variants are particularly rare and can often present with attenuated phenotypes compared to non-mosaic cases (summarized in Table 1).

**Table 1.** Reported germline and mosaic STAG2 variants and associated clinical phenotypes in patients with STAG2-related cohesinopathies.

Reference (Year, Journal)	STAG2 Variant	Variant Type	Sex / Age	Clinical Features
Kong et al., 2025 (16)	c.3222dup p.Ser1075IlefsTer12	Frameshift	F / 8 years	Bilateral panretinal congenital hypertrophy of the retinal pigment epithelium (CHRPE), optic disc pallor, STAG2-related phenotype without major neurodevelopmental impairment, microcephaly, speech delay, ADHD
Mullegama et al., 2018 (17)	c.3027A>T p.Lys1009Asn	Missense	M / 4 years	Developmental delay, undergrowth/failure to thrive, microcephaly, facial dysmorphism, polydactyly
Mullegama et al., 2017 (18)	c.205C>T p.Arg69Ter	Nonsense	Mostly females, pediatric patients	Global developmental delay, microcephaly, hypotonia, facial dysmorphism, multiple congenital anomalies, neurodevelopmental impairment
Mullegama et al., 2017 (18)	c.1811G>A p.Arg604Gln	Missense		
Mullegama et al., 2017 (18)	c.1913_1922del p.Ala638ValfsTer10	Frameshift		
Kruszka et al., 2019 (19)	c.3034C>T p.Arg1012Ter	Nonsense	Newborn	Holoprosencephaly, severe brain malformations, craniofacial anomalies associated with cohesin complex defects including STAG2
Kruszka et al., 2019 (19)	c.205C>T p.Arg69Ter	Nonsense	2 years	
Kruszka et al., 2019 (19)	c.436C>T p.Arg146Ter	Nonsense	32 weeks	
Kruszka et al., 2019 (19)	c.2533+1G>A	Splice-site	Newborn / deceased	
Kruszka et al., 2019 (19)	c.2898_2899del p.Glu968SerfsTer15	Frameshift	1 year	
Kruszka et al., 2019 (19)	c.775C>T p.Arg259Ter	Nonsense	9,5 years	
Yuan et al., 2018 (20)	c.418C>T p.Gln140Ter	Nonsense	F / 3 years	Variable cohesinopathy phenotype including developmental delay, intellectual disability, growth abnormalities, and congenital malformations
Yuan et al., 2018 (20)	c.1605T>A p.Cys535Ter	Nonsense	F / 4 years	
Yuan et al., 2018 (20)	c.1811G>A p.Arg604Gln	Missense	F / 11 years	
Yuan et al., 2018 (20)	c.1658_1660delinsT p.Lys553IfsTer6	Frameshift	F / 1 year	
Yuan et al., 2018 (20)	c.476A>G p.Tyr159Cys	Missense	M / 5 years	
Freyberger et al., 2021 (21)	p.Tyr159His	Missense	M / 11 years	Neonatal hypotonia, developmental delay, intellectual disability, facial dysmorphism, neurological and craniofacial abnormalities; expanded MKMS phenotype
Duff et al., 2025 (22)	c.1196+4_1196+7del	Splice-site	F / Pediatric	Expanded MKMS phenotype including central diabetes insipidus and acute kidney injury
Aoi et al., 2020 (23)	c.3097C>T p.Arg1033Ter	Nonsense	M / Newborn	Distinct congenital anomalies, developmental delay, microcephaly, facial dysmorphism, multisystem involvement
Aoi et al., 2020 (23)	c.2229G>A p.Trp743Ter	Nonsense	F / 7 years	
Schmidt et al., 2022 (24)	c. 2184G>T p.Gln728>His	Missense	Multiple patients; both males and females, pediatric	Mosaic STAG2 variants associated with developmental delay, microcephaly, intellectual disability, facial dysmorphism, limb anomalies, growth retardation, congenital malformations, and variable severity depending on mosaic distribution
Schmidt et al., 2022 (24)	c.1412_1416+9del	Splice-site		

CHRPE = Congenital Hypertrophy of the Retinal Pigment Epithelium; MKMS = Mullegama-Klein-Martinez Syndrome; STAG2 = Stromal Antigen 2; ID = Intellectual Disability; DD = Developmental Delay

To the best of our knowledge, the co-occurrence of GATA2 deficiency with probable constitutional STAG2 mosaicism has not previously been reported. Here, we describe a case characterized by a heterozygous germline GATA2 truncating variant and a STAG2 frameshift variant with molecular and clinical findings supporting probable constitutional mosaicism.

The patient presented with clinical features suggestive of GATA2 deficiency, but also with dysmorphic features consistent with the phenotypic spectrum of MKMS. Molecular testing across different tissues confirmed the germline GATA2 variant and provided additional evidence supporting probable constitutional mosaicism involving STAG2. This case highlights the importance of broad molecular testing in patients with atypical or overlapping phenotypes and raises the hypothesis that co-occurring constitutional alterations affecting distinct regulatory mechanisms may converge on pathways relevant to hematopoietic dysfunction and myeloid disease predisposition.

## Methods

### Patient ascertainment

This study was conducted in compliance to the principles of the Helsinki Declaration and Institutional Review Board (IRB) and approved by the ethics committee of the Fundeni Clinical Institute. Written informed consent was obtained from the patient prior to inclusion in the study, including consent for genetic investigations, publication of clinical data, and use of anonymized images for scientific purposes. Comprehensive clinical evaluation included review of the patient's medical history, hematological and biochemical investigations, bone marrow studies, neurological assessment, imaging findings, and clinical genetic examination.

In addition, peripheral blood samples were obtained from the proband’s mother for molecular and cytogenetic analyses in order to investigate potential familial segregation.

### Bionformatics tools

Variant annotation and interpretation were performed using publicly available databases and bioinformatic resources, including ClinVar, gnomAD, DECIPHER, Ensembl, and UniProt/Swiss-Prot.

Exploratory structural modeling of STAG2 was performed using the SWISS-MODEL web-based protein structure modeling platform. The wild-type amino-acid sequence corresponding to the MANE Select transcript NM_001042750.2 was retrieved from Ensembl. The p.Phe834Leufs*38 sequence was generated from the reference sequence by incorporating the frameshift-derived amino-acid sequence followed by the premature termination codon, and the wild-type and hypothetical truncated sequences were modeled independently. For the wild-type sequence, the highest-ranked template identified by SWISS-MODEL was the AlphaFold v2 model B5FW45.1.A (99.29% sequence identity; GMQE 0.78). For the hypothetical truncated sequence, the selected template was the experimentally determined cryo-EM structure of cohesin subunit SA-2 within the SA2-SCC1 complex (9hmv.1.A; 2.9 Å resolution; 97.66% sequence identity; target coverage 0.98). The resulting truncated model covered residues 83–854 and yielded a GMQE of 0.83 and a global QMEANDisCo score of 0.84 ± 0.05. Given the predicted susceptibility of the mutant transcript to nonsense-mediated mRNA decay (NMD), the truncated structural model was interpreted exclusively as an exploratory representation of a hypothetical translation product in the event of NMD escape and residual translation, and not as evidence for the production of a stable truncated protein *in vivo*.

## Results

### Case presentation

A 25-year-old male was referred to our hematology department for a second-opinion evaluation of suspected hypocellular myelodysplastic syndrome (MDS) or an inherited bone marrow failure, in the context of persistent cytopenias and recurrent infectious complications. During childhood, the patient had been clinically diagnosed with Emberger syndrome based on unilateral lower-limb lymphedema, chronic pancytopenia, and cutaneous abnormalities, without molecular confirmation. He was born to non-consanguineous parents of Romanian ancestry, and the family history was unremarkable for hematologic malignancies, congenital disorders, recurrent pregnancy loss, or other hereditary conditions.

His recent medical history was notable for two septic episodes within the preceding year, including a generalized cutaneous staphylococcal infection and a pulmonary infection, both requiring antibiotic treatment and followed by worsening cytopenias. He also had a history of psoriasis treated with acitretin and prednisone, complicated by staphylococcal infection. Hematologically, persistent mild pancytopenia was documented, including neutropenia ranging approximately between 1.0 and 1.3 × 10^3^/µL, without a chronic transfusion requirement. During septic episodes, thrombocytopenia (30–50 × 10^3^/µL) and severe anemia (hemoglobin 60–70 g/L) were recorded. Abdominal ultrasonography revealed mild splenomegaly (13.6 cm longitudinal diameter), and bone marrow hypoplasia had previously been diagnosed.

At first presentation to our center, laboratory evaluation showed leukopenia (2.8 × 10^3^/µL), thrombocytopenia (77 × 10^3^/µL), and a hemoglobin level of 11.4 g/dL. Peripheral blood smear demonstrated anisopoikilocytosis and granulocytic dysplasia, including hypogranulation and hypolobulated nuclei. No folate or vitamin B12 deficiency was identified, and serum haptoglobin was within the normal range.

Bone marrow aspirate was hypocellular and showed a decreased granulocytic lineage (40%) with dysplastic features, including hypogranulation and hypolobulation, and 1% myeloblasts. The erythroid lineage was relatively expanded (42%–44% erythroblasts) and showed normoblastic maturation. The megakaryocytic lineage was scarcely represented. Reactive mature lymphocytes and plasma cells were present but did not exceed 10%. Hemosiderin deposits and plasmacytosis were noted, without ring sideroblasts. Bone marrow biopsy confirmed reduced cellularity (∼35%) with increased adipose tissue. All hematopoietic lineages were represented; the erythroid lineage showed normoblastic maturation, whereas the granulocytic lineage was reduced but retained maturation. Micromegakaryocytes were very rare, including forms with hypolobulated nuclei, and CD34-positive cells accounted for approximately 1%. Overall, the findings were consistent with bone marrow hypoplasia with megakaryocytic involvement. According to the WHO 5th edition classification, the hematologic disease was classified as myelodysplastic neoplasm with low blasts (MDS-LB), associated with germline GATA2 predisposition. According to the 2022 International Consensus Classification (ICC), the findings were consistent with MDS, NOS, in the context of germline GATA2 predisposition. The IPSS-M score was −0.41, corresponding to the Moderate Low risk category. Given the underlying hereditary predisposition, the IPSS-M result was considered complementary prognostic information and interpreted with caution.

Additional laboratory findings included elevated serum erythropoietin (306 mIU/mL; reference range, 4.3–29 mIU/mL) and ferritin (458.8 ng/mL; reference range, 30–300 ng/mL). Peripheral blood lymphocyte immunophenotyping showed weak CD56 positivity without evidence of a defined lymphoproliferative disorder.

**Figure 1:**
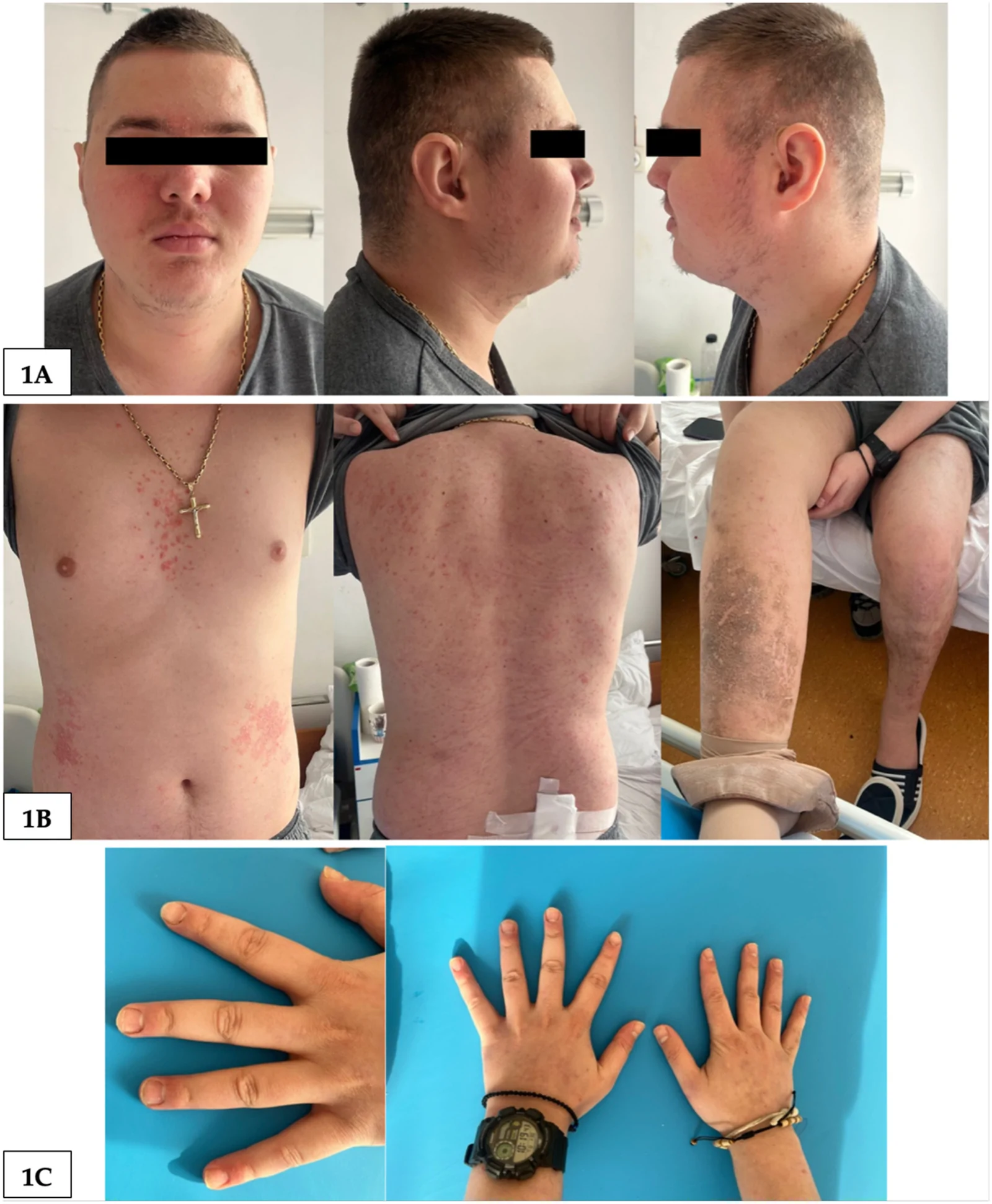
Clinical findings (**(1A)** dolichocephaly, midface hypoplasia, epicanthus, microstomia with thick lips, malar hypoplasia, short bulbous nose, high forehead; **(1B)** psoriasis lesions; lymphedema; **(1C)** brittle hair and nails and deeply set nails).

At presentation, the patient exhibited unilateral lower-limb lymphedema, generalized psoriatic skin lesions, onychomycosis, and progressive hearing loss. Genetic examination revealed multiple qualitative dysmorphologic features, including coarse facial features, a narrow forehead, dolichocephaly, midface hypoplasia, epicanthus, microstomia with thick lips, malar hypoplasia, a broad nasal bridge, a short bulbous nose, a high forehead, high palate, dental abnormalities, brittle hair and nails, and mildly hypoplastic, flattened, deeply set nail plates (Figure 1). Overall growth and head size were clinically unremarkable, and no additional anthropometric abnormalities were identified during genetic examination. The clinically assessed phenotypic findings were standardized using Human Phenotype Ontology (HPO) terminology, where applicable, and are summarized in Supplementary Table 1. Because dysmorphism is not a typical feature of GATA2 deficiency, further evaluation was performed. Electroencephalography and brain MRI showed no abnormalities, and psychological evaluation confirmed normal intellectual development.

Conventional cytogenetic analysis of bone marrow aspirate demonstrated a normal male karyotype (46,XY). Fluorescence *in situ* hybridization (FISH) analysis for 7q deletion was negative. Multiplex ligation-dependent probe amplification (SALSA MLPA Probemix P414-C1 MDS, MRC Holland) did not reveal copy number alterations involving MDS-related loci (the genomic loci interrogated are provided in Supplementary Table 2).

Targeted Next-Generation Sequencing (Oncomine Myeloid DNA Panel, Thermo Fisher Scientific) from peripheral blood identified three pathogenic variants: STAG2 NM_001042750.2: c.2502delC (p.Phe834Leufs*38), variant allele frequency (VAF) 38.76%; GATA2 NM_032638.5: c.1084C > T (p.Arg362Ter), VAF 50.65%; and ASXL1 NM_015338.6: c.1934_1935insG (p.Gly646Trpfs*12), VAF 43.52% (Supplementary Table 3). All three were truncating variants with well-established pathogenicity in myeloid malignancies. According to the 2017 AMP/ASCO/CAP guidelines for somatic variant interpretation, all were initially classified as Tier IA (clinically significant).

However, the near-heterozygous allelic fractions (∼50%) observed for both the GATA2 and STAG2 variants raised suspicion of a germline origin. Subsequent next-generation sequencing (NGS) analysis performed on buccal swab-derived DNA confirmed the presence of the GATA2 p.Arg362Ter and STAG2 p.Phe834Leufs*38 variants, both detected at approximately 50% variant allele frequency (VAF). The higher VAF observed in buccal-derived DNA may reflect tissue-specific distribution of the mosaic event and/or variable representation of mutant cellular populations. In contrast, the ASXL1 c.1934_1935insG (p.Gly646Trpfs*12) variant was not detected in buccal-derived DNA, supporting its acquired somatic origin and its interpretation as a secondary clonal event. This interpretation was also consistent with the clinical phenotype, as constitutional loss-of-function ASXL1 variants are associated with Bohring-Opitz syndrome, a clinically distinct neurodevelopmental disorder, whereas our patient had normal intellectual development and lacked the characteristic syndromic presentation.

The GATA2 variant was subsequently classified according to germline variant interpretation criteria, based on the American College of Medical Genetics and Genomics 2015 guidelines, with subsequent updates (ACGS 2024). The STAG2 variant was also evaluated using germline criteria in the context of the suspected constitutional mosaicism, while acknowledging that its constitutional origin could not be definitively established (Supplementary Table 4).

Maternal testing was performed primarily because STAG2 is X-linked and the proband is male, in order to assess whether the STAG2 alteration, if constitutional, could have been maternally inherited. The STAG2 variant was not detected in the maternal sample, and therefore the available findings do not support maternal inheritance. The GATA2 variant was also not detected in the mother, as GATA2 was included in the same assay. Also, neither parent exhibited clinical features suggestive of GATA2 deficiency or a STAG2-related disorder. Paternal testing was not performed; consequently, a *de novo* origin of the GATA2 variant could not be established.

The STAG2 variant required additional evaluation given its X-linked localization and the approximately 50% VAF observed in buccal-derived DNA from a male patient. Fluorescence *in situ* hybridization (FISH) analysis of the X chromosome demonstrated the presence of a single X chromosome in all analyzed metaphases, excluding sex-chromosome aneuploidy as an explanation for the observed allelic fraction. Together with the detection of the STAG2 variant in buccal-derived DNA and the presence of an overlapping STAG2-related phenotype, these findings provided additional evidence supporting probable constitutional mosaicism. However, because buccal specimens may contain hematopoietic cells, confirmation in an independent non-hematopoietic tissue would have been required to definitively establish constitutional mosaicism. Such additional sampling was not available. Therefore, although the combined clinical, molecular, and cytogenetic findings support probable constitutional STAG2 mosaicism, an acquired hematopoietic origin cannot be completely excluded.

### Clinical management and follow-up

Following completion of the second-opinion hematologic and genetic evaluation, the patient returned to the local hematology center responsible for his ongoing care. Based on the hematologic findings and the confirmed germline GATA2 predisposition, evaluation for hematopoietic stem cell transplantation was recommended. Subsequent clinical follow-up was conducted outside our institution; therefore, information regarding further disease evolution, completion of transplant evaluation, donor selection, or eventual transplantation was not available. As the patient was no longer under follow-up at our center, additional sampling for confirmation of the STAG2 finding in an independent non-hematopoietic tissue could not be performed.

### Exploratory bioinformatic analyses supporting functional convergence

**Figure 2:**
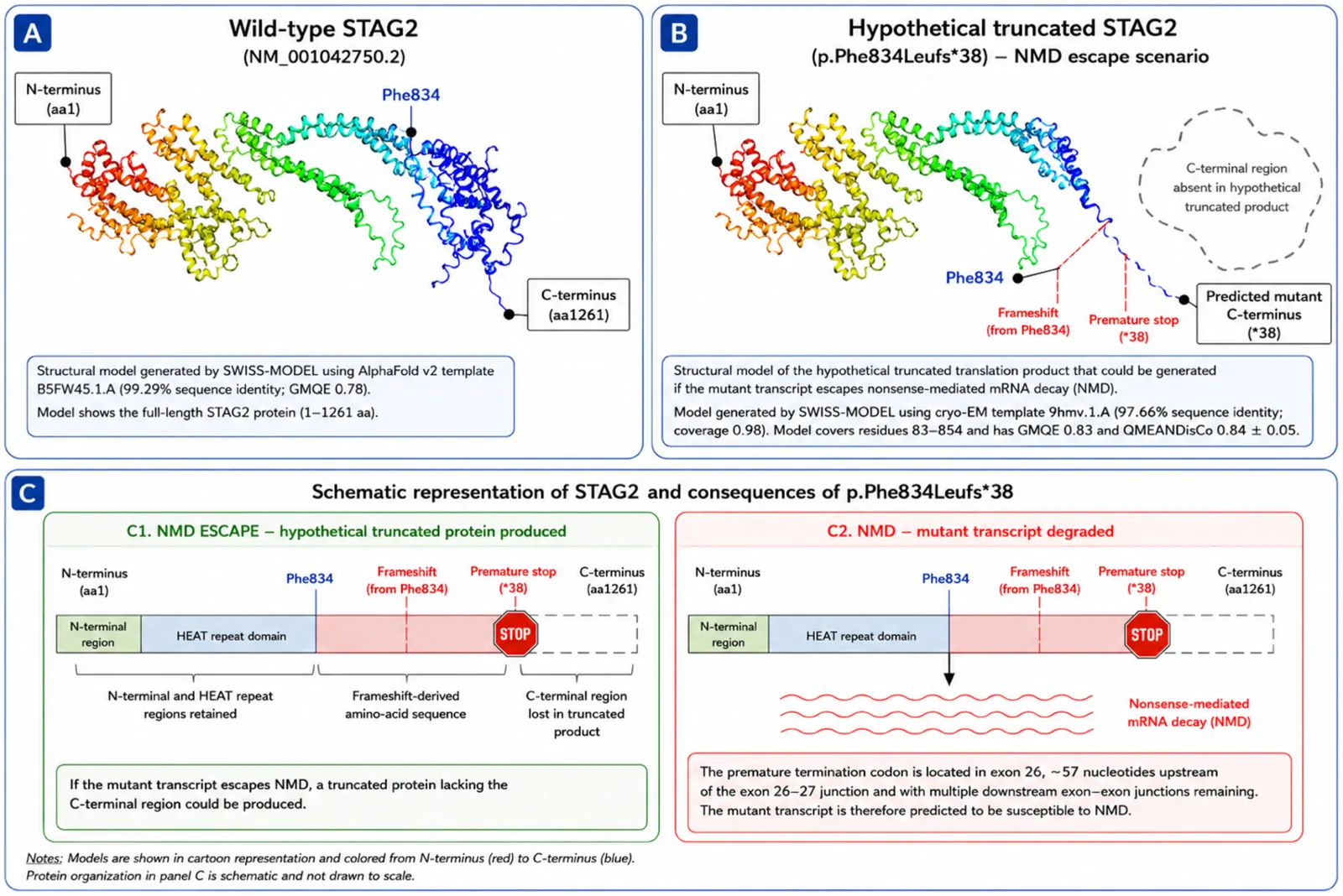
Exploratory structural modeling and predicted molecular consequences of the STAG2 p.Phe834Leufs*38 variant. **(A)** Predicted 3D structure of wild-type STAG2. **(B)** Structural model of the hypothetical truncated p.Phe834Leufs*38 translation product in the event of NMD escape. **(C)** Schematic representation of the two potential molecular outcomes of the frameshift: (C1) NMD escape with generation of a hypothetical C-terminally truncated protein, and (C2) degradation of the mutant transcript through NMD. The premature termination codon is located in exon 26 of NM_001042750.2, approximately 57 nucleotides upstream of the exon 26–27 junction, predicting susceptibility to NMD. Domain organization is shown schematically and is not drawn to scale.

In silico analysis of the STAG2 p.Phe834Leufs*38 variant was performed to explore its predicted molecular consequences (Figure 2). The frameshift alters the amino-acid sequence from residue Phe834 and introduces a premature termination codon after 38 codons in the altered reading frame. The premature termination codon is located in exon 26 of transcript NM_001042750.2, approximately 57 nucleotides upstream of the exon 26–27 junction, with multiple downstream exon-exon junctions remaining. According to the canonical 50–55 nucleotide rule, the mutant transcript is therefore predicted to be susceptible to nonsense-mediated mRNA decay (NMD), representing the most plausible primary loss-of-function mechanism.

Structural modeling was additionally performed as an exploratory visualization of the potential protein-level consequences in the event of NMD escape and residual translation. Comparison with wild-type STAG2 illustrated the substantial loss of the C-terminal structural region in the hypothetical truncated product. The model was not interpreted as evidence that a stable truncated protein is produced *in vivo*, and local conformational differences distant from the frameshift site were not considered evidence of a specific pathogenic mechanism. Rather, the analysis illustrates the extent of structural loss that could result if the mutant transcript escapes NMD and undergoes translation (24, 25).

## Discussion

This case illustrates how myelodysplastic syndrome (MDS) in young adults may occur in the context of an underlying constitutional predisposition that can remain incompletely characterized without extended germline investigation. In our patient, a clinical diagnosis of Emberger syndrome had been considered during childhood based on lymphedema, chronic cytopenias, and cutaneous manifestations, but had never been molecularly confirmed. At referral to our center, the combination of persistent cytopenias, recurrent severe infections, hypocellular marrow involvement, and atypical dysmorphic features prompted a comprehensive molecular evaluation for an inherited hematologic predisposition. This ultimately confirmed germline GATA2 deficiency and identified an additional STAG2 alteration with molecular and clinical findings supporting probable constitutional mosaicism. To our knowledge, the co-occurrence of germline GATA2 deficiency with probable constitutional STAG2 mosaicism in a patient with MDS has not previously been reported.

GATA2 deficiency represents a well-established hereditary predisposition syndrome associated with bone marrow failure, immunodeficiency, and increased lifetime risk of myeloid malignancies, particularly MDS and AML. However, affected individuals may present with variable and sometimes incomplete phenotypes, and a syndromic diagnosis based exclusively on clinical findings may remain uncertain until molecular confirmation is obtained. Our findings further emphasize that variants initially detected through somatic testing in hematologic malignancies may in fact reflect underlying constitutional predisposition and therefore warrant evaluation in non-hematopoietic tissue whenever clinically indicated. This distinction is particularly important in young patients presenting with cytopenias, hypocellular marrow, unusual infectious history, or syndromic features (26, 27).

While somatic STAG2 alterations are frequently identified during clonal evolution in myeloid neoplasms, constitutional STAG2 involvement remains rare and is predominantly described in the context of cohesinopathies and neurodevelopmental disorders. In the present case, detection of the STAG2 variant in buccal-derived DNA, together with the attenuated dysmorphic phenotype, absence of significant neurodevelopmental impairment, and exclusion of sex-chromosome aneuploidy, provided converging evidence supporting probable constitutional mosaic STAG2 involvement. However, because buccal specimens may contain hematopoietic cells and confirmation in an independent non-hematopoietic tissue was not available, an acquired hematopoietic origin cannot be completely excluded. Accordingly, the STAG2 finding is interpreted as probable, rather than definitively demonstrated, constitutional mosaicism. Importantly, the presence of atypical dysmorphic features not usually associated with isolated GATA2 deficiency represented a major clue prompting broader genetic investigation (13, 15).

An alternative interpretation that must be considered is that the STAG2 alteration represents an acquired event arising during clonal evolution of GATA2-associated MDS, similar to the concomitant ASXL1 alteration. Both STAG2 and ASXL1 are recurrent somatic alterations in GATA2-deficient myeloid disease. In the present case, however, their tissue distribution differed: both variants were detected at substantial VAFs in peripheral blood, whereas subsequent analysis of buccal-derived DNA detected the STAG2 variant but not the ASXL1 variant. This discordant distribution provides additional support for a constitutional contribution of STAG2 rather than simply assuming that both alterations represent components of the same acquired hematopoietic clone. Nevertheless, because hematopoietic contamination of the buccal specimen cannot be completely excluded, an acquired STAG2 alteration remains a possible alternative interpretation. Accordingly, the available findings support, but do not definitively establish, probable constitutional STAG2 mosaicism.

From a biological perspective, the coexistence of GATA2 and STAG2 alterations raises the hypothesis that disruption of distinct but potentially complementary mechanisms may contribute to the hematopoietic phenotype observed in this patient. GATA2 is a transcriptional regulator essential for hematopoietic stem and progenitor cell maintenance, whereas STAG2 is a core component of the cohesin complex involved in chromatin organization, sister chromatid cohesion, and genome architecture. Although these established functions provide biological plausibility for a potential interplay between GATA2 deficiency and STAG2 dysfunction, the present single-case observation does not demonstrate functional cooperation, synergy, or causality. Such a relationship remains hypothetical and would require dedicated functional studies for validation (3, 9, 28).

An additional notable finding was the presence of a secondary somatic ASXL1 truncating alteration, a well-recognized marker of clonal evolution and adverse prognosis in myeloid malignancies. This observation is consistent with the dynamic nature of disease progression in patients with inherited predisposition syndromes and highlights the importance of longitudinal molecular monitoring in such cases. Taken together, the molecular findings are compatible with a multi-hit model in which an underlying constitutional predisposition is accompanied by secondary acquired alterations during clonal evolution; however, the relative contribution of each alteration to disease development cannot be established from a single case (3).

This case also carries important clinical implications. In the present case, integration of the hematologic and genetic findings led to a recommendation for hematopoietic stem cell transplantation evaluation. Because subsequent care was provided outside our institution, we cannot determine whether transplantation was ultimately pursued or whether the molecular findings subsequently influenced donor selection. In particular, related donor selection requires careful evaluation in order to avoid the use of asymptomatic carriers of pathogenic predisposition variants. Moreover, the present report reinforces the importance of integrating clinical genetics into the evaluation of young adults presenting with unexplained cytopenias, hypocellular MDS, or atypical multisystemic manifestations (3, 29, 30).

Several limitations should nevertheless be acknowledged. Confirmation of the STAG2 alteration in an independent non-hematopoietic tissue was not feasible, limiting our ability to definitively establish its constitutional origin, the timing of the presumed mosaic event, and its tissue distribution. Although detection of the variant in buccal-derived DNA supports constitutional involvement, variable hematopoietic contamination of buccal specimens cannot be completely excluded. Accordingly, the STAG2 alteration is regarded as probable constitutional mosaicism rather than definitively confirmed constitutional mosaicism.

Consequently, the mechanistic relationship between the identified alterations and the development of myelodysplastic features should be interpreted with caution. As this report describes a single patient, conclusions regarding genotype-phenotype correlations, functional interaction, or synergistic pathogenic mechanisms cannot be established. Nevertheless, the rarity of the observed molecular combination and the integrated clinical-genetic findings provide a rationale for further investigation of the potential contribution of co-occurring constitutional alterations to the phenotypic and hematologic variability observed in inherited myeloid predisposition syndromes.

### Practical implications for molecular diagnostics

This case illustrates several diagnostic pitfalls that may arise during the molecular evaluation of young patients with myelodysplastic syndromes. First, pathogenic variants identified through somatic NGS panels may be incorrectly assumed to represent acquired alterations, particularly when they involve genes frequently mutated in myeloid neoplasms, such as GATA2 or STAG2. In the present case, both variants were initially interpreted as clinically significant somatic findings according to current AMP/ASCO/CAP recommendations, potentially overlooking an underlying constitutional predisposition. The combination of the patient's young age, syndromic clinical features, and unexpected variant allele frequencies prompted additional tissue-specific molecular investigation, which confirmed the germline origin of the GATA2 variant and provided evidence supporting probable constitutional mosaicism involving STAG2.

Second, constitutional mosaicism represents an additional diagnostic challenge. In male patients, an unexpected variant allele frequency for an X-linked gene may be misinterpreted as technical variability or clonal hematopoiesis unless mosaicism is actively considered. In the present case, integration of cytogenetic findings, detection of the STAG2 variant in buccal-derived DNA, and the overlapping clinical phenotype provided converging evidence supporting probable constitutional mosaic STAG2 involvement. However, because buccal specimens may contain hematopoietic cells and confirmation in an independent non-hematopoietic tissue was not available, an acquired hematopoietic origin cannot be completely excluded. Finally, this case emphasizes that molecular interpretation should never rely exclusively on sequencing results. Accurate classification requires integration of clinical genetics, dysmorphology, family history, cytogenetics, and, whenever feasible, confirmation in an independent non-hematopoietic tissue when constitutional mosaicism is suspected.

## Conclusions

In conclusion, this case highlights the importance of distinguishing between somatic and constitutional variants in young patients presenting with cytopenias or myelodysplastic features. Unexpected variant allele frequencies, particularly when accompanied by syndromic or multisystemic features, should prompt consideration of an underlying constitutional predisposition and, when appropriate, additional tissue-specific molecular investigation. In the present case, this approach confirmed germline GATA2 deficiency and provided converging evidence supporting probable constitutional mosaic STAG2 involvement, while the ASXL1 alteration was consistent with a secondary acquired event. Accurate interpretation requires integration of molecular findings with clinical phenotype, family history, cytogenetics, and tissue-specific analyses, as misclassification may affect genetic counseling, surveillance, and clinical management.

As comprehensive NGS panels become increasingly integrated into routine hematology practice, multidisciplinary interpretation will be essential to recognize inherited predisposition syndromes within apparently somatic molecular profiles. This case further illustrates the diagnostic value of comprehensive molecular evaluation in patients with atypical or overlapping phenotypes and emphasizes that constitutional mosaicism should be considered when molecular findings cannot be fully reconciled with the clinical and cytogenetic context.

## Data Availability

The original contributions presented in the study are included in the article/Supplementary Material, further inquiries can be directed to the corresponding authors.
