# Supplementary material for: Co-occurrence of GATA2 deficiency and probable constitutional mosaic STAG2 alteration in a young adult with myelodysplastic syndrome: a case report
Source: Front Med (Lausanne). 2026 Sep 2;13:1935947. doi: 10.3389/fmed.2026.1935947 (PMC13577846; doi:10.3389/fmed.2026.1935947)
Supplement: Supplementary file 1 [file Table1.docx]

# **Supplementary_Table 1. Standardized phenotypic features of the patient according to Human Phenotype Ontology (HPO) terminology**

| Clinical finding | HPO standardized term | HPO ID |
| --- | --- | --- |
| Coarse facial features | Coarse facial features | HP:0000280 |
| Narrow forehead | Narrow forehead | HP:0000341 |
| Dolichocephaly | Dolichocephaly | HP:0000268 |
| Midface hypoplasia | Midface retrusion | HP:0011800 |
| Epicanthus | Epicanthus | HP:0000286 |
| Microstomia with thick lips | Narrow mouth | HP:0000160 |
| Malar hypoplasia | Malar flattening | HP:0000272 |
| Broad nasal bridge | Wide nasal bridge | HP:0000431 |
| Short bulbous nose | Bulbous nose | HP:0000414 |
| High forehead | High forehead | HP:0000348 |
| High palate | High palate | HP:0000218 |
| Dental abnormalities | Abnormality of the dentition | HP:0000164 |
| Brittle hair and nails | Brittle hair  Fragile nails | HP:0002299  HP:0001808 |
| Mildly hypoplastic, flattened, deeply set nail plates | Mildly hypoplastic, flattened, deeply set nail plates | Descriptive finding; no specific HPO term assigned |

# **Supplementary_Table 2. SALSA MLPA Probemix P414-C1 MDS**

| **Length**  **(nt)** | **SALSA MLPA probe** | **Chromosomal position (hg18)** | | | **Location (hg18) in kb** |
| --- | --- | --- | --- | --- | --- |
|  |  | Reference | Target | Mutation-specific |  |
| 118 | **ZFY probe** S0135-L13810 |  | Yp11.31 |  | Y-002,889 |
| 122 | Reference probe 19616-L27299 | 4p13 |  |  | 04-042,278 |
| 127 | **MIR145 probe** 19041-L29241 |  | 5q33.1 |  | 05-148,790 |
| 133 | **IKZF1 probe** 03340-L20689 |  | 7p12.2 |  | 07-050,338 |
| 138 | **NCOA2 probe** 09938-L20198 |  | 8q13.3 |  | 08-071,250 |
| 143 | Reference probe 14199-L15813 | 2q13 |  |  | 02-108,894 |
| 151 | **AATF probe** 21021-L29242 |  | 17q12 |  | 17-032,463 |
| 155 | **RPS14 probe** 16292-L18584 |  | 5q33.1 |  | 05-149,807 |
| 160 | **MYC** **probe** 20780-L29239 |  | 8q24.21 |  | 08-128,822 |
| 167 | **TP53** **probe** 01588-L06028 |  | 17p13.1 |  | 17-007,531 |
| 172 | Reference probe 08726-L25038 | 9q21 |  |  | 09-078,038 |
| 176 | **SRC** **probe** 22671-L19475 |  | 2q11.23 |  | 20-035,448 |
| 182 | **ETS1** **probe** 09496-L25025 |  | 11q24.3 |  | 11-127,836 |
| 186 | **KMT2A** **probe** 19885-L29240 |  | 11q23.3 |  | 11-117,898 |
| 192 | **SAMD9L** **probe** 16293-L24868 |  | 7q21.2 |  | 07-092,600 |
| 197 | **KMT2A** **probe** 17085-L24869 |  | 11q23.3 |  | 11-117,853 |
| 202 | **PTK2** **probe** 18560-L24870 |  | 8q24.3 |  | 08-141,739 |
| 208 | **JAK2** **probe** 05672-L17742 |  |  | **p.V617F (c.1849G>T)** | 09-005,064 |
| 214 | **ASXL1** **probe** 18515-L25024 |  | 2q11.21 |  | 20-030,480 |
| 220 | **SUZ12** **probe** 18518-L23809 |  | 17q11.2 |  | 17-027,345 |
| 227 | Reference probe 05282-L31924 | 14q22 |  |  | 14-050,130 |
| 232 | **SPARC** **probe** 16294-L24866 |  | 5q33.1 |  | 05-151,047 |
| 238 | **EPO** **probe** 17089-L20201 |  | 7q22.1 |  | 07-100,158 |
| 244 | **MET** **probe** 10323-L10837 |  | 7q31.2 |  | 07-116,199 |
| 250 | **MLH1** **probe** 18556-L24871 |  | 3p22.2 |  | 03-037,031 |
| 256 | **TP53** **probe** 02376-L24176 |  | 17p13.1 |  | 17-007,519 |
| 263 | Reference probe 14738-L20398 | 4q22 |  |  | 04-089,187 |
| 268 | **MIR146A** **probe** 15653-L24279 |  | 5q33.3 |  | 05-159,845 |
| 274 | **CDKN1B probe** 07949-L24280 |  | 12p13.1 |  | 12-012,762 |
| 282 | **EZH2 probe** 18271-SP0635-L23385 |  | 7q36.1 |  | 07-148,135 |
| 286 | **EGR1 probe** 22557-L32018 |  | 5q31.2 |  | 05-137,829 |
| 294 | Reference probe 04570-L25187 | 16q13 |  |  | 16-055,491 |
| 301 | **EGR1 probe** 17091-L20203 |  | 5q31.2 |  | 05-137,832 |
| 308 | **EZH2 probe** 22673-L23019 |  | 7q36.1 |  | 07-148,145 |
| 315 | **SPARC probe** 19042-L25092 |  | 5q33.1 |  | 05-151,027 |
| 324 | Reference probe 22672-L26274 | 18q21 |  |  | 18-045,659 |
| 332 | **TIRAP probe** 18557-L25022 |  | 11q24.2 |  | 11-125,666 |
| 341 | **NF1 probe** 02507-L25021 |  | 17q11.2 |  | 17-026,576 |
| 348 | **RUNX1T1 probe** 09487-L24873 |  | 8q21.3 |  | 08-093,099 |
| 355 | **ZMYND8 probe** 14661-L16313 |  | 2q13.12 |  | 20-045,308 |
| 361 | **CDK6 probe** 18558-L02523 |  |   19q13.42 |  | 07-092,085 |
| 368 | **PRPF31 probe** 06016-L29420 |  |  |  | 19-059,319 |
| 377 | Reference probe 10693-L19115 | 6p12 |  |  | 06-051,721 |
| 385 | **FGFR1 probe** 01046-L24278 |  | 8p12 |  | 08-038,434 |
| 392 | **ETV6 probe** 13875-L19638 |  | 12p13.2 |  | 12-011,797 |
| 400 | **NIPBL probe** 04837-L24177 |  | 5p13.2 |  | 05-037,039 |
| 409 | Reference probe 13405-L31765 | 6q12 |  |  | 06-065,393 |
| 415 | **HNF4A probe** 09999-L29451 |  | 2q13.12 |  | 20-042,477 |
| 421 | **APC probe** 01807-L29244 |  | 5q22.2 |  | 05-112,201 |
| 427 | **KMT2E probe** 18796-L29272 |  | 7q22.2 |  | 07-104,490 |
| 436 | Reference probe 08839-L32008 | 2p13 |  |  | 02-071,767 |
| 445 | **MECOM probe** 18573-L24179 |  | 3q26.2 |  | 03-170,332 |
| 454 | **TP53 probe** 08785-L19640 |  | 17p13.1 |  | 17-007,515 |
| 463 | **SMARCA4 probe** 09980-L10439 |  | 19p13.2 |  | 19-011,000 |
| 472 | Reference probe 00979-L31258 | 10p14 |  |  | 10-012,019 |
| 481 | **GATA2 probe** 18576-SP0668-L23908 |  | 3q21.3 |  | 03-129,685 |
| 490 | **ETV6 probe** 13871-L24874 |  | 12p13.2 |  | 12-011,914 |
| 496 | Reference probe 09772-L25949 | 15q21 |  |  | 15-042,706 |

**Supplementary_Table 3. Somatic variant interpretation from peripheral blood (ASCO/AMP/CAP 2017)**

| Gene | Nucleotide change | Protein change | VAF | Effect | Classification |
| --- | --- | --- | --- | --- | --- |
| STAG2 | c.2502delC | p.F834Lfs*38 | 38.76% | Loss of function, truncating | Tier IA |
| GATA2 | c.1084C>T | p.R362Ter | 50.65% | Loss of function, nonsense | Tier IA |
| ASXL1 | c.1934_1935insG | p.G646Wfs*12 | 43.52% | Loss of function, frameshift | Tier IA |

**Supplementary_Table 4. Germline reclassification after buccal swab testing (ACMG/AMP criteria)**

| Gene and locus | Variant | Variant type | Status | Inheritance | Classification |
| --- | --- | --- | --- | --- | --- |
| *GATA2* (3q21.3) | c.1084C>T (p.R362Ter) | Nonsense substitution | Germline heterozygous (VAF 52.6%) | Autosomal dominant | Pathogenic (PVS1, PM1, PM2, PM6) |
| *STAG2* (Xq25) | c.2502delC (p.F834Lfs*38) | Frameshift deletion | Germline mosaic (VAF 68.05%) | X-linked | Pathogenic (PVS1, PS2, PM2) |
